# Framework of the outreach after a school shooting and the students perceptions of the provided support

**DOI:** 10.3402/ejpt.v5.23079

**Published:** 2014-07-02

**Authors:** Tuija Turunen, Henna Haravuori, Jaakko J. Pihlajamäki, Mauri Marttunen, Raija-Leena Punamäki

**Affiliations:** 1 Hospital District of South Ostrobothnia, Seinäjoki, Finland; 2 Department of Psychology, School of Social Sciences and Humanities, University of Tampere, Tampere, Finland; 3 Department of Mental Health and Substance Abuse Services, National Institute for Health and Welfare, Helsinki, Finland; 4 Department of Psychiatry, Helsinki University Central Hospital, Helsinki, Finland; 5 Department of Adolescent Psychiatry, Helsinki University Central Hospital, Helsinki, Finland

**Keywords:** School shooting, psychosocial support, trauma, youth, bereaved families

## Abstract

**Background:**

A large number of bereaved family members, surviving students, and their relatives as well as school staff and the wider community were in need of psychosocial support as a result of a school shooting in Kauhajoki, Finland, 2008. A multilevel outreach project provided psychosocial care to the trauma-affected families, students, schools staff, and wider community for 2 years and 4 months.

**Objective:**

This article is twofold. First, it presents the theoretical rationale behind the psychosocial support and describes the multimodal elements of the services. Second, it analyzes the trauma-exposed students’ help-seeking behavior and perceptions of the usefulness of the support they were offered in different phases of recovery.

**Method:**

Information of students’ help-seeking and perceptions of support is based on a follow-up data from 4 months (T1, *N*=236), 16 months (T2, *N*=180), and 28 months (T3, *N*=137) after the shootings. Mean age of students was 24.9 (SD=10.2; 95% women). Their perceptions of the offered psychosocial support were collected with structured and open questions constructed for the study.

**Results:**

The results confirmed the importance of enhancing the natural networks after a major trauma and offering additional professional support for those in greatest need. The students’ perceptions of the provided care confirmed that the model of the acute and long-term outreach can be used after major tragedies in diverse situations and in other countries as well.

The accumulated knowledge about short- and long-term consequences of a mass trauma is incorporated in several evidence-based and evidence-informed guidelines and consensus statements for psychosocial care after disasters (Call, Pefferbaum, Jenuwine, & Flynn, 2012; Hobfoll et al., 2007; NICE, 2005; Pfefferbaum, Shaw, & AACAP, 2013; TENTS, 2008). The guidelines emphasize both promoting resilience and treating prolonged psychological distress after traumatic events and systematic planning and management of care. They also argue for the usefulness of specific elements of interventions in immediate, acute, and ongoing phases of recovery. In the early- to mid-term stages of mass trauma aftercare, the aim is to locate the most vulnerable and needy and to provide information and psychoeducation in order to promote survivors’ sense of safety, to calm down hyperarousal, and to facilitate feelings of belongingness and community efficacy (Hobfoll et al., 2007).

Support and services should be available for both families and individuals, and the interventions should be based on assessed physical, psychological, and social needs of the recipients. Psychoeducation provides balancing effects, information, and assurance; topics can include common reactions to trauma, access to services, and self-help methods (TENTS, 2008). According to the guidelines, in the later phases of recovery, the provided care involves more therapeutic elements and is tailored according to survivors’ and families’ unique needs. When psychotherapy is used, Trauma Focused Cognitive Behavioral Therapy and Eye Movement Desensitization and Reprocessing (EMDR) are prioritized (TENTS, 2008; World Health Organization [WHO], 2013).

Activating the survivors’ natural support systems is one of the primary aims for professional aftercare, as social support has been found to be a major protective factor in the recovery process (Brewin, Andrews, & Valentine, 2000). The timing and nature of survivors’ responses and mental health problems differ, and therefore the emphasis is on the long-term tailored care and interventions even for several years (Hobfoll et al., 2007; TENTS, 2008). After a shooting incident, the school is a natural environment to provide psychosocial support to trauma-affected students and to identify those in need for intensive support (Pfefferbaum et al., 2013). Rescue workers and health care professionals are under intensive stress after mass trauma such as a school shooting and outreach programs should include prevention of vicarious traumatization (Galea, Nandi, & Vlahov, 2005; TENTS, 2008).

## Kauhajoki school shooting

In September, 2008, a student of Seinäjoki University of Applied Sciences entered the school building in Kauhajoki armed with a hand gun and opened fire indiscriminately. He shot to death nine of his classmates and a teacher and threatened several others. He also set fires and damaged the premises. Other students and the school staff managed to escape from the building (Ministry of Justice [MOJ], 2010). The majority of the students were females aged between 15 and 25. At the time of the shooting, there were approximately 260 students and 40 staff members inside the school.

The emergency situation following the shooting lasted several hours in the town of Kauhajoki and every school in the vicinity was alerted. The students were kept inside their school buildings for several hours, because of the potential danger. Malicious threats via SMS-messages toward other schools in the South Ostrobothnia area spread quickly, as did rumors of possible new massacres. Subsequently other school communities also experienced the terror caused by the massacre. Their need for psychosocial support was also acknowledged. The tragedy was overwhelming for the police, rescue workers, health care professionals, and other authorities, and they needed extra supervision and support.

## Aims of the study

There is little research about the ways of delivering theory-based psychosocial care after mass trauma, and about recipients’ experiences of the provided support. The aim of this article was twofold:

1. To describe the framework of a multilevel outreach model, which provided psychosocial care to the families of the deceased, students, and school staff, as well as the wider community in the aftermath of the school shooting tragedy (part 1).
2. To analyze the surviving students’ help-seeking behavior and their perceptions of the usefulness and the healing elements of the multi-level support (part 2).

### Part 1: Implementation of an outreach model

#### Preparation, management, and organizing crisis help

Every municipality in Finland is obliged to offer psychosocial first aid and support after catastrophes and disasters. This activity is commonly arranged by the local crisis teams, for example, with psychologists, general practitioners, and social workers with expertise in traumatic stress. The local crisis teams are, however, intended for providing only the immediate and acute support. As the need for long-term support was anticipated after the school shooting, a multidisciplinary project was founded. The aim of the outreach was to ensure that all traumatized persons and groups would have access to psychosocial support according to their needs and phases of recovery (Ala-aho & Turunen, 2012; Turunen & Punamäki, 2014). Table 1 presents examples of the psychosocial support provided to the families of the deceased, students, school staff, and the wider community in the immediate, acute, later, and ongoing phases of recovery.

**Table 1:** The main elements of the psychosocial support provided to families, students, and school staff according to the level of interventions and phase of exposure and recovery

	Families of the deceased		Students and staff exposed to the shootings	
		
Level of intervention	Immediate and acute phase	Later and ongoing recovery	Immediate and acute phase	Later and ongoing recovery
Individual	• Services of the crisis clinic • Support when visiting the scene of the massacre • Practical assistance	• Services of the crisis clinic • Psychotherapies • Physiotherapies • Practical assistance	• Services of the crisis clinic • Interviews to assess the severity of exposure and available support	• Services of the crisis clinic • Interviews to assess the need of extra support among the most severely exposed • Screening of the possible posttraumatic reactions at 2, 4, 16, and 28 months • Health check-ups, medical assessment • Psychotherapies • Physiotherapies and massage
Family	• Group discussions • Support for families visiting the scene of the massacre • Telephone contact with every family to ensure the sufficiency and appropriateness of support	• Frequent contacts by telephone to assess the unique needs of each family member • Two home visits to assess the family situation and needs • Support in emotionally demanding occasions	• Family evenings at the school	• Professionally led peer support group process
Group	• Information about the services provided by the Kauhajoki Project • Letter providing psychoeducative information and an invitation to join the peer support group process	• Professionally led peer support group process • Support in emotionally demanding situations • Rituals	• Group discussions separately for the staff and students • Common sessions with psychoeducation and rituals	• Group discussions separately for the staff and students • Supervision sessions for teachers • Rituals
Community			• Services of the crisis clinic • Group discussions in the other schools at the area • Parents**'** evenings in the other schools at the area • Media coverage with psychoeducative and calming content	• Services of the crisis clinic • Reinforced youth work and student welfare • Comprehensive media coverage around the first anniversary • Open doors at the trauma-affected school after moving back to the premises

#### Implementing psychosocial care at immediate and acute phases

The recipients of the immediate support were the evacuated students, school staff, and families searching for their loved ones, as well as other citizens in shock. The interventions included helping families to connect with their children, providing facts regarding the situation, and giving information about the services that were available for them. Furthermore, they involved monitoring overwhelming and incontrollable trauma reactions, and providing support and medical assessment for those in need. An outpatient crisis clinic provided services 24 hours a day for the first 2 weeks and, ultimately, during office hours. A telephone hotline with health care specialists answering questions was open during the first days, and a website was launched for crisis support and information.

#### Support for the families of the deceased

The relatives of the deceased were a target group for psychosocial support, grief counseling, and practical assistance. They were provided guidance, information, and psychoeducation about common responses to trauma and helpful coping. Additional psychosocial support was available for the families in the emotionally charged occasions, such as visiting the scene of the massacre, respecting anniversaries, and attending trials. Psychotherapy was offered to family members who were in need for it according to the clinical assessments, and professionally led peer support group process was used as a group intervention for all the families of the deceased at the ongoing phases of recovery. The families were offered five peer support gatherings over 2 years. These weekend-long gatherings consisted of psychoeducative lectures, peer discussions, joint evening programs, as well as rituals for longing and recovery (Turunen & Punamäki, in press). The family of the perpetrator also received psychotherapeutic support, and a separate group process.

#### Support for the students and school staff

The psychosocial support and services for the trauma-affected school were embedded in the school community's everyday life in order to make the access to services as easy as possible. The action plan was developed and implemented in close cooperation with the administration and staff of the school. Participation in all services was voluntary. The phase model of the support provided to the trauma-affected students and staff is summarized in Turunen & Punamäki (2014).

Individual support was proactively offered especially to those who had a severe trauma exposure and/or strong reactions. Common sessions for the whole school community were conducted daily for the first week to offer practical information, psychoeducation, and joint activities. Similar sessions were arranged whenever increasing of trauma-related stress was anticipated, that is, moving back to the renovated school, releasing police reports, and the first anniversary.

Group discussions with psychoeducative content were offered to students and staff. The groups gathered initially a couple of days after the shootings, and three to six times during the mid-term and ongoing recovery stages. The groups were led by a crisis psychologist and a psychiatric nurse. The psychoeducation involved teaching stress management techniques, normalizing of stress reactions, and general knowledge of trauma consequences. In the staff groups topics included also how the trauma may have an impact on academic performance and how the teachers may help the students to regulate heightened emotional arousal. According to the principle of watchful waiting (NICE, 2005) posttraumatic stress symptoms (PTSS) were screened by health care specialists and a research group at 2, 4, 16, and 28 months. Students and staff exceeding clinically significant levels of symptoms were referred to therapeutic services. Teachers were also offered supervision.

A professionally led peer support group was also conducted as a group intervention for the most severely exposed students and their family members. It contained three 1-day-long workshops with psychoeducative information; peer group discussions for parents, siblings, and students; and a visit to the school when the renovation was completed. The first meeting took place 3 months after the tragedy, the second around the first anniversary, and the last around the second anniversary.

#### Psychosocial services at the community level

Aftercare services at the community level were carried out in cooperation with the local authorities such as youth work and the management of the schools. The school shooting also had an impact on the students in the other schools in the area and the student welfare systems were therefore reinforced in several school units. The media was used as a means to provide information to the citizens. The information was psychoeducative in nature, and aimed at promoting parenting resources, normal routines, and social support.

### Part 2: Surviving students’ help-seeking behavior and their perceptions of the usefulness and the healing elements of the multi-level support

#### Method

##### Participants and procedure

Experiences of the exposed students were collected as a part of a 2-year follow-up study carried out by the National Institute for Health and Welfare. The basic sample was 389 students of the exposed school, who were approached 4 months after the shooting. The actual participants were 236 students (60.7% response rate) at 4 months after the shooting (T1). One-fifth of the basic sample (20.1%; *n*=78) declined and another fifth could not be reached (19.5%; *n*=76). The mean age of the participants was 24.9 (*SD*=10.2), and the majority were females (95%). The students participated again at 16 months’ (T2, *n*=180) and 28 months’ (T3, *n*=137) follow-up. The study protocol was accepted by the ethics committee of the Hospital District of South Ostrobothnia. Participation was voluntary and every participant was asked to sign a written informed consent. The first and second assessments were carried out in the school and the third follow-up questionnaire was posted to the participants. The participating students who reported high levels of PTSS or other psychological distress were referred to the outreach services.

##### Measures

**The severity of trauma exposure** was based on the degree of threat to life and suffered losses. At T1, the students answered yes or no to 19 questions concerning their experiences during the school shootings (e.g., “I lost a friend/friends,” “I had to escape the perpetrator,” or “I saw someone to get shot”). The answers were categorized into five classes according to the severity of the exposure including categories of “mild, moderate, significant, severe, and extreme exposure” (Suomalainen et al., 2011). “Mild exposure” was rated when the student was not at the building at the time of the shootings. “Moderate exposure” was rated when a student evacuated from the building without being in a direct life danger and did not lose any acquaintances. “Significant exposure” was when a student had to act to escape the shooter, had to hide to avoid a danger to life, saw bodies, or lost acquaintances. Exposure was considered “Severe” when a student was near mortal danger, saw somebody threatened with a gun, or lost someone significant. When the exposure was rated as “Extreme” a student had been in a mortal danger or saw someone being shot or lost a family member. For the analysis, a dichotomy variable was formed: (1) Severely to extremely exposed students, and (2) Mildly to significantly exposed students.

**The use of immediate crisis support** was assessed by four questions at T1: whether the student was offered crisis support immediately after the incident irrespective of the provider (yes/no), whether they had accepted and used any of the services (yes/no), and whether they had attended the sessions for the whole school community (yes/no). Finally, students were asked about their perceptions about the usefulness of the immediate crisis support using a 5-point scale: 1=helped a lot, 2=helped enough, 3=helped a little, 4=did not help, and 5=hindered recovery. Reporting 1 or 2 was recorded as immediate crisis support being helpful, whereas 3, 4, and 5 was recorded as immediate crisis support not being helpful.

**The use of psychosocial support at the acute, later, and ongoing phases** was assessed with 13 questions on the source and availability of support in all assessment points T1, T2, and T3. The sources of support were grouped as *social support from families and friends* (family, other relatives, friends), *professional support* (crisis workers for the school community, use of low-threshold crisis clinic, municipal health care center, student health care and/or psychiatric outpatient clinics), and *social support from others* (teachers, youth workers, workers of the parish, clubs, or extracurricular activities). Concerning the availability of different types of support, the students estimated whether they had received (1) no support, (2) some support, (3) enough support, (4) too much support, or (5) had not been interested in the provided support. Reporting “too much” or “enough” support was rated as having the support available.

The perceived effect of the different types of psychosocial support were evaluated with five alternative answers (1) did not help, (2) cannot say, (3) did help, (4) was irritating, and (5) not interested. Answering “did help” was indicative for perceiving the support helpful while the other alternative answers were indicative for support not being helpful. Students were also asked if they had started psychotherapy or regular meetings with health care professionals and whether or not psychotherapy included EMDR. Students answered yes or no to these questions. The students were also asked about the time when they had started psychotherapy.

**Students’ perceptions of the professional support and its healing elements** were studied with two open questions. Students answered at T1, T2, and T3 to questions: “Where did you get the most important help for your traumatic and distressing experiences?” and “What was the most important reason for its healing effect.” The answers indicating professional support as being helpful were selected for further analysis. Two coders (a clinician and a researcher) classified the answers to the question “What was the most important reason for its healing effect” in 10 categories according to the themes of the answers. The 10 categories were then reclassified into five final categories, which represent the concepts of psychosocial support. The coders classified the answers separately and deviating scores were settled by consensus.

## Statistical analyses

Distributions of the use and perception of psychosocial services in immediate and acute phase were presented as percentages for categorical variables and as means (M) and standard deviations (SD) for continuous variables. Differences between the groups (e.g., with different exposure severity) were tested using the chi-square tests and analyses of variance. In the analyses, two-tailed significance levels <.05 were chosen. All analyses were performed using SPSS 20.0.

## Results

### Students’ perception of the psychosocial support

Table 2 presents the use and perceptions of the different types of psychosocial support in the immediate, acute, later, and ongoing phases of recovery. A majority of the students (84.7%) had been offered immediate crisis support within the first 24 hours after the events and 58.5% of them accepted the support. Almost all of the students (92.4%, *n*=110) who accepted the support estimated that the support had helped them “a lot” or “enough.” Furthermore, more than two-thirds of the students attended the common sessions for the whole school during the first week and more than half attended the group sessions.

**Table 2:** Psychosocial support and care, and therapies for the students of the exposed school

	All students	Severely to extremely exposed students	Mildly to significantly exposed students	
				
Type of the support	*T1: n*=236 *n* (%)^a^	*n*=20^a^ *n* (%)^a^,^b^	*n*=216^a^ *n* (%)^a^,^b^	Difference between the exposure groups
Immediate crisis support^c^				
Reached by immediate (first 24 hours) crisis support	199 (84.7)	20 (100.0)	179 (89.9)	*χ* ^2^=3.96, df=1, *p*=.047
Immediate crisis support accepted	113 (58.5)	15 (75.0)	98 (56.6)	n.s.
Perceived accepted immediate crisis support as helpful	110 (92.4)	15 (100.0)	95 (91.3)	n.s.
Group and school sessions				
Attended the common sessions for the whole school	167 (71.1)	17 (85.0)	150 (69.8)	n.s.
Attended the group sessions	140 (60.6)	18 (90.0)	122 (57.8)	*χ* ^2^=7.92, df=1, *p*=.005
Acute phases psychosocial support^c^				
From families and friends	232 (98.7)	20 (100.0)	212 (98.6)	n.s.
From others	179 (79.6)	15 (78.9)	164 (79.6)	n.s.
From Professionals	164 (71.0)	18 (90.0)	146 (69.2)	*χ* ^2^=3.84, df=1, *p*=.050
Perceived the received crisis support as helpful				
Families and friends (T1)	220 (97.8)	19 (95.0)	201 (98.0)	n.s.
Others (T2)	148 (89.2)	14 (93.3)	134 (88.7)	n.s.
Professionals (T1)	114 (78.6)	12 (75.0)	102 (79.1)	n.s.
Professionals (T2)^d^	83 (89.2)	11 (91.7)	72 (88.9)	n.s.
Professionals (T3)^e^	76 (73.1)	11 (91.7)	65 (70.7)	n.s.
Psychotherapy or regular meetings^f^ T1-, T3	60 (25.4)	13 (65.0)	47 (21.8)	*χ* ^2^=18.05, df=1, *p*<.001
Psychotherapy included EMDR T1-T3	12 (20.0)	6 (46.2)	6 (12.8)	*p*=.015, exact

n.s=not significant.

a Valid percentages shown (missing data not included).

b Percentages shown within the exposure group.

c Crisis support after the first day and within 2 weeks after the incident, availability of support asked by different sources.

d Answers to the question about perception of professional support at T2 (16 months follow-up), *n*=123 within those who have received the services.

e Answers to the question about perception of professional support at T3 (28 months follow-up), *n*=104 within those who have received the services.

f Shows cumulative numbers and percentages across T1 to T3.

Concerning the severity of exposure to school shooting, all students with severe to extreme exposure to trauma had received the immediate support, which statistically differed from those with less severe exposure (*p*<.05). There was no significant difference in perception of the helpfulness of the accepted immediate psychosocial support according to the severity of the trauma as reported at T1. Similarly, students with severe to extreme exposure to trauma used more professional psychosocial support than the less severely exposed in both the acute and ongoing phases of recovery (*p*<.001). The type of support involved mostly psychotherapy or regular meetings with health care professionals. One-fifth (20%) of the psychotherapies included EMDR-therapy as well. A majority of the students who were offered professional help perceived it helpful at a later phase (89%) and (73%) at ongoing phase of recovery as reported in T2 and T3. The perceptions did not differ according to the severity of the exposure to school shooting trauma.

Table 3 presents students’ perceptions of the support at the acute phase. It reveals that students predominantly relied on their natural social relations for support. They mentioned family members (57%), and friends and peers (54%) equally often as the main sources of support, assistance, and consolation. They accounted that family support enhanced their sense of safety and affiliation and felt at ease in sharing the pain with the family members. The helpfulness of peers and friends as support persons was based on sharing of similar feelings of horror, uncertainty, and common experiences of fear of death.

**Table 3:** Sources of the support among the students exposed to the school shootings in acute phase (T1): who provided the most important help and what was perceived as healing element(s)

Main source of the support	*n*=236 *n* (%)	Healing elements	Examples
Own family and close relatives	134 (56.8)	• Intimacy • Love	• Intimacy and speaking about normal daily life issues • Mother and her genuine concern and love • I have the best dad in the world
Friends and fellow-students	127 (53.8)	• Peer support • Understanding because of similar experience	• It is easiest to talk to the close persons you can trust • Just being close, total presence, and feeling of understanding without words
Teachers and other school staff	14 (5.9)	• Togetherness • Understanding because of similar experience	• The best help comes from people who had experienced the same tragedy • We feel attached to our school, and that helps us
Crisis psychologists, psychiatrists, and other professionals	61 (25.8)	• Sharing the story • Professionalism • Psychoeducation • Therapeutic interventions • Enhancing safety	• Sessions with the psychiatrist consisted of real listening and deep understanding, not only of being together • The crisis psychologist listened, supported, and forwarded to the medical doctor • Crisis workers provided information about how to cope and how to deal with normal daily life issues and what helps you to continue your life • The groups in which we were together, that was a decisive experience in recovery • The awareness that there are crises workers available if needed, that has helped me
Church and parish	6 (2.5)	• Spiritual consolation	• My own parish and belonging to it, I was allowed to share and leave my worries to God
None or I cannot say	18 (7.6)		• I know that there was all kind of help available. But I did not have time to go, and also the strangeness of others does not help

*Note*: The percentages do not sum up to 100.0 because students mentioned more than one source of support and reasons as healing elements.

About a quarter of the students evaluated professional help as helpful at the acute phase, reported at T1, 4 months after the shooting. The most healing elements were practical assistance, psychoeducation, and creating of therapeutic alliance and emotional transference. Students perceived that the organized aftercare helped them to feel more secure. Teachers also served as a source of assistance and condolence, and created a feeling of stability for the trauma-affected students, and 6% of them perceived that as helpful. The parish and church were considered helpful (3%) as they provided shelter, a possible place to gather together, and to enjoy silence and individual support.

Table 4 summarizes the healing elements of professional care that the students perceived most helpful at the ongoing stages of recovery. They reported them at 16 (T2) and 28 months (T3) after the school shooting. More than a half of the recipients regarded the opportunity to narrate, frame, and share their frightening experiences as being beneficial. The proactive attitudes and emotional support from professionals were considered helpful, and students also emphasized the usefulness of psychoeducation and stress management. They mentioned examples such as “how to breathe and calm yourself” or “she gave permission to the emotions I considered to be crazy.” Furthermore, they emphasized the relevance of continuity of the services (same providing professionals) and specific therapeutic interventions (medication and psychotherapeutic methods). The students felt that the professionals enhanced the feeling of safety (“Where ever I met them I immediately felt safe”).

**Table 4:** The helpful elements of the professional support reported by students of the exposed school at ongoing recovery phases at T2 (16 months) and T3 (28 months) afterwards

Helpful element	T2 *n*=42 *n* (%)	T3 *n*=35 *n* (%)
Sharing the story • Forming the narrative, listening, supporting	22 (52.4)	20 (57.1)
Professionalism • Expertise, neutrality, active support	9 (21.4)	13 (37.1)
Psychoeducation • Normalizing, teaching self-care techniques	6 (14.3)	9 (25.7)
Therapeutic interventions • Group interventions, therapeutic relationship • Medication/EMDR	3 (7.1)	5 (14.3)
Enhancing safety, continuity • Creating feeling of safety • Stability of the professionals	2 (4.8)	6 (17.1)

*Note*: The percentages do not sum up to 100.0 because students mentioned more than one element of support as being helpful. Only answers with argumentation were classified.

## Discussion

In mass trauma situations, the need for psychological support is enormous and provision of services should start immediately, yet bearing in mind that the most important source of support for the traumatized is the support given by their natural networks. Professional care can supplement the natural social support by offering psychoeducation, support, and treatment in an active but discreet manner, promoting resiliency. The tailored services described here were provided via multilevel outreach, which followed the national and international guidelines, best practices, and consensus statements of acute, mid-term, and long-term psychosocial support after disasters.

The students’ feedback, which is analyzed in this study, shows that they found the availability of psychosocial support helpful. The important role of intimate networks in enhancing recovery concurs with earlier studies that are conducted among school shooting survivors (Littleton, Grills-Taquechel, & Axsom, 2009; Murtonen, Suomalainen, Haravuori, & Marttunen, 2012). Almost 99% of the exposed students in Kauhajoki received support from family, relatives, or friends and almost all perceived it helpful. This is in line with the attachment theory revealing that the early created attachment system activates in the face of threat and distress, and the traumatized individuals seek comfort and safety from their close social relationships (Bowlby, 1969/1982; Mikulincer & Shaver, 2010, p. 12). Accordingly, the guidelines point out family members and other natural networks as the most important source of support for the traumatized survivors (Hobfoll et al., 2007; TENTS, 2008). The role of professional support is to facilitate activation of these natural networks, to offer psychoeducation and support, as well as to screen for those whose natural networks’ support fails, whose trauma-related distress is severe, or who otherwise are at high risk for PTSD or other psychological impairment (Hobfoll et al., 2007; Pfefferbaum et al., 2013; TENTS, 2008).

The psychosocial support was offered to the families of the deceased, and the students and staff immediately after the tragedy, and it was extensively and proactively offered especially for those who were in greatest need as is recommended (Call et al., 2012; Hobfoll et al., 2007; Pfefferbaum et al., 2013; TENTS, 2008). The acute help for the trauma-affected students and staff included several psychoeducative group discussions and common sessions. They provided practical information, assurance for safety, and psychoeducation about acute stress responses. Constructing a coherent and shared narrative about the trauma is important as it is suggested to facilitate recovery from trauma in ongoing phases (Shaw, 2000).

Trauma-related symptoms may be delayed in occurrence, and the readiness to seek and receive support varies between individuals (Bonnano, 2004; Turunen, Haravuori, Punamäki, Suomalainen, & Marttunen, in press). Therefore “watchful waiting” principle was applied (NICE, 2005; TENTS, 2008) in order to be ready for potential delayed PTSS and re-evoked needs for psychosocial support. Professional support was especially targeted to the most severely exposed students, and most of them evaluated the support as helpful in all phases of recovery. Students appreciated the stability and continuity of aftercare services, and the neutrality and professional expertise of their familiar crisis workers. They expressed positive views on learning about common trauma-related responses, effective coping, and other ways of regulating arousals and stress. Frequent screening turned out to be a helpful tool for monitoring the progress of recovery process, and the professional interventions and intensive support could be allocated and targeted to those suffering from psychological distress.

The follow-up showed that students who were most severely exposed to the shooting were common clients in psychotherapy. One-fifth of the psychotherapies included also EMDR-therapy, which is a recommended treatment in various guidelines (Duodecim, 2009; TENTS, 2008; WHO, 2013). As a conclusion, the students’ perceptions of the provided professional support were mainly positive, which indicates the usefulness of the outreach.

The study can be criticized for drop-out, retrospective setting for the students’ experiences, and narrowness of descriptive data. The lack of systematic collection of experiences and opinions of other trauma-affected survivors such as family members or school staff is unfortunate. The study could reach 60.7% of the trauma-exposed students at 4 months (T1) after the school shootings, indicating reasonably high response rate in the field of trauma study. The loss of participants was not associated with the severity of trauma exposure. It may have been difficult for the students to assess in retrospect the quality of the acute services. Ethically, however, the 4 months as a baseline for the follow-up study was well chosen. The results of both structured and open questions are coherent, and support each other. The students’ short responses to the open questions do not naturally depict in depth their experiences of the traumatization, psychosocial support and recovery. For that a qualitative research method would be more fitting.

## Conclusion

The access to the psychosocial services needs to be easy after a tragedy that affects a large number of citizens. Support and care should be available for long enough time. The positive perceptions of the interventions provided within this outreach model suggest that like models may be used in other situations and countries after a mass traumatic event.
